# Flexible PET/ITO/Ag SERS Platform for Label-Free Detection of Pesticides

**DOI:** 10.3390/bios9030111

**Published:** 2019-09-19

**Authors:** Ariadna B. Nowicka, Marta Czaplicka, Aneta A. Kowalska, Tomasz Szymborski, Agnieszka Kamińska

**Affiliations:** Institute of Physical Chemistry, Polish Academy of Sciences, Kasprzaka 44/52, 01-224 Warsaw, Poland; anowicka@ichf.edu.pl (A.B.N.); mczaplicka@ichf.edu.pl (M.C.); anetakowalska29@gmail.com (A.A.K.); akaminska@ichf.edu.pl (A.K.)

**Keywords:** surface-enhanced Raman spectroscopy (SERS), dielectric barrier discharge, flexible SERS platform, pesticide, Thiram, Carbaryl

## Abstract

We show a new type of elastic surface-enhanced Raman spectroscopy (SERS) platform made of poly(ethylene terephthalate) (PET) covered with a layer of indium tin oxide (ITO). This composite is subjected to dielectric barrier discharge (DBD) that develops the active surface of the PET/ITO foil. To enhance the Raman signal, a modified composite was covered with a thin layer of silver using the physical vapor deposition (PVD) technique. The SERS platform was used for measurements of *para*-mercaptobenzoic acid (*p*-MBA) and popular pesticides, i.e., Thiram and Carbaryl. The detection and identification of pesticides on the surface of fruits and vegetables is a crucial issue due to extensive use of those chemical substances for plant fungicide and insecticide protection. Therefore, the developed PET/ITO/Ag SERS platform was dedicated to quantitative analysis of selected pesticides, i.e., Thiram and Carbaryl from fruits. The presented SERS platform exhibits excellent enhancement and reproducibility of the Raman signal, which enables the trace analysis of these pesticides in the range up to their maximum residues limit. Based on the constructed calibration curves, the pesticide concentrations from the skin of apples was estimated as 2.5 µg/mL and 0.012 µg/mL for Thiram and Carbaryl, respectively. Additionally, the PET/ITO/Ag SERS platform satisfies other spectroscopic properties required for trace pesticide analysis e.g., ease, cost-effective method of preparation, and specially designed physical properties, especially flexibility and transparency, that broaden the sampling versatility to irregular surfaces.

## 1. Introduction

Pesticides are chemical substances (natural or synthetic) that are used to prevent, destroy or control harmful organisms (as well as disease and weeds), or to protects plants or plant products during production, storage and transport [1,2]. The pesticides can be classified into two groups: first, synthetic, which is further classified into five classes: organochlorine, organophosphate, carbamate, neonicotinoid, and pyrethroid. The second group is biopesticides, which contain microbial pesticides (e.g., bacteria, entomopathogenic fungi, viruses), biochemical pesticides, herbal pesticides, plant-incorporated protectants and RNAi pesticides [3]. There are more than 1000 active substances worldwide that can be used in pesticides, with sales of over 2.5 million tons [1]. According to the European Environment Agency/Eurostat there are more than 500 active substances that can be used in pesticides in the European Union, and over 350,000 tons are sold there every year. The main area of application for these pesticides are agricultural production, horticulture and forestry [4]. The pesticides are toxic to pests and therefore, they are also a threat to the general environment (e.g., groundwater and soils) [5,6], wildlife (e.g., reduction of biodiversity) [7] and human beings (e.g., gastrointestinal, neurological, and carcinogenic risks) [8,9]. For these reasons, monitoring and detecting the pesticides and their residues is extremely important for the environment and the food industry. Because of the high sensitivity and reproducibility, various techniques based on chromatographic methods (LC/GC-MS, HPLC and TLC) are used for detection of pesticides [10,11]. These methods are expensive and time consuming, therefore new methods that are cheaper and faster, with similar sensitivity and reproducibility, are needed.

One of the most promising methods for fast and precise detection and identification of pesticides is surface-enhanced Raman spectroscopy (SERS) [3,11,12,13] and the first attempts to detect pesticides via the SERS method was performed by Alak and Vo-Dinh [14]. SERS is a non-destructive technique, that is highly sensitive, selective, reliable and allows for fast detection of samples. The phenomenon of SERS is described by the combination of an electromagnetic mechanism (EM) and a chemical mechanism (CT) related to charge transfer between an adsorbate and substrate. The electromagnetic enhancement results from the amplification of light by excitation of surface plasmon resonance (SPR) of the substrate. Of the two processes, the EM mechanism usually plays a larger role in SERS enhancement. Theoretically, the electromagnetic enhancement factor (EF) can reach 10^3^–10^11^, whilst chemical enhancement factors up to 10^3^ were calculated [15]. This huge enhancement of Raman scattering (even single molecules can be observed) ensures that SERS spectroscopy is used in many areas, for example in medicine [16], environmental analysis [17], pharmaceuticals [18], food [19], and industry [20].

SERS detection is fast, reliable and can go down to a single molecule, which makes it an ideal technique for detection of pesticides. However, to enhance the signal, this method needs a special SERS platform, which is usually made of silicon, glass or another brittle material. The residues of pesticides are located on the curved surface of the fruits and vegetables, therefore materials with a high Young’s modulus are not the best choice. For this reason, SERS platforms based on elastic materials (e.g., elastomers, fabrics and polymers) have been used in recent years for detection of chemical residues on the surface of food (fruits, vegetables or even fish). This is also the main reason why flexible SERS platforms for detection of pesticides have become very popular. Such flexible SERS platforms are characterized by various properties:

(i) They are easy to fabricate: easy to bend and cut to any shape (in contrast to substrates based on silicon, glass or quartz),
(ii) They do not crumble and/or break at loads with bending and torsional moments,
(iii) They offer high enhancement of the Raman signal, so they can be used in most analytical applications,
(iv) The technological process is simple and finished or semi-finished products (e.g., films coated with conductive oxide, foils or fabrics) can be purchased commercially in large quantities,
(v) Flexible SERS platforms can be attached to rough, irregular surfaces and can be used to take samples directly from the surface of a body or pesticides from the surface of fruit (“paste and peel off” approach),
(vi) Due to their flexibility, they offer increased versatility of sampling, e.g., swabs or micro-extractions,
(vii) They are easy to use in the field (no need to apply solution as in the case of colloids or nanoparticles).

The most commonly used flexible SERS platforms are paper [21,22], filter paper [23], fabrics [24,25,26], poly(dimethylsiloxane) (PDMS) films [27,28,29], fiber cloth [30], electrospun fibers [31], surface-modified polymers [32,33] and also egg shell membrane [34], modified adhesive tape [35,36], sandpaper [37], copper foil [38] and cardboard [39]. Also, successful measurements have been performed using modified cicada and dragonfly wings [40,41,42,43]. The methods of modifications include both top-down and bottom-up techniques: physical vapor deposition (PVD) [44], electrodeposition [45], electrospinning [46], Forcespinning™ [47], chemical etching [48], ink-jet printing [49], dip-coating [50] and others [51]. SERS platforms are also used for the detection of small molecules and biomaterials [52,53,54,55] and the flexible SERS platform seems to be a promising tool for diagnostics [56,57,58].

Corona discharge is a widely known and used method of modification of the surfaces of polymer films and foils. A popular variant of this method is dielectric barrier discharge (DBD) [59,60]. DBD is also known as silent discharge or atmospheric-pressure-glow discharge and was described for the first time in 1857 by Ernst Werner von Siemens. The DBD method ensures easy formation of stable plasma, it is easy to scale up and to use as it can be performed under atmospheric pressure. It involves a high voltage power supply connected to two electrodes: one electrode has high potential, whereas the second is grounded. At least one dielectric barrier, typically plastic, mica, glass or another insulator, is placed between these electrodes to prevent arcing. In most applications (modification of the surface of the polymers and fibers) DBD is applied with the use of a kHz alternating electric field, however in past years ranges higher than 1 MHz have been used [61]. The role of dielectric in DBD is crucial: Once the discharge occurs between the electrodes, the charge is transported towards the grounded electrode and accumulated at the surface of the dielectric. The charge reduces the electric field in the discharge gap and interrupts the flow of the current. The length of this pulse of current depends on the pressure, characteristics of effective ionization of the gas and dielectric properties of the isolator. Therefore, in DBD the AC electric field produces a large number of micro-discharges, which are randomly distributed both in time and in space between electrodes. The dielectric layer has two functions: (i) to limit the amount of the charge transported within a single micro-discharge and (ii) to distribute the micro-discharges uniformly between the electrodes [61].

In this paper, we demonstrate a new, flexible type of SERS platform based on commercially available poly(ethylene terephthalate) (PET) covered with a layer of indium tin oxide (ITO) (PET/ITO) foil, which was subjected to DBD. The silent discharge modifies the surface of the foil, develops and makes it rougher. Covering such a modified PET/ITO foil with a SERS-active metal (e.g., Au or Ag) makes it an effective, flexible SERS platform. Additionally, the possibility of using of an elaborated flexible SERS platform is presented by detecting Thiram, and Carbaryl pesticides in apple juice. The presented platform is easy and fast to manufacture, offers high EF and can be used for detection of pesticides in both the laboratory and the field.

## 2. Materials and Methods

### 2.1. Materials

The initial material for the SERS platform was poly(ethylene terephthalate) (PET) foil covered with indium tin oxide (ITO). Sheets of a PET/ITO composite were generously given by Southwall Europe GmbH (Großröhrsdorf, Germany). The foil was thermally stabilized PET DuPont Melinex ST504 with 125 µm thickness and covered with 5 µm layer of ITO. 

*Para*-mercaptobenzoic acid (*p*-MBA) was a chemical compound used as a standard for platform optimization, and pesticides (i.e., Thiram and Carbaryl, see Figure S1 in Supplementary Materials for their chemical structure) were applied in all experiments. All were purchased from Sigma-Aldrich (Dorset, UK) and used without further purification. 

Millipore water was used throughout the experiments. The organic fruits used in the experiments were obtained from the local apple planter. They were rinsed with deionized water and dried at room temperature before further use.

### 2.2. Preparation of the SERS Platform

**Figure 1:**
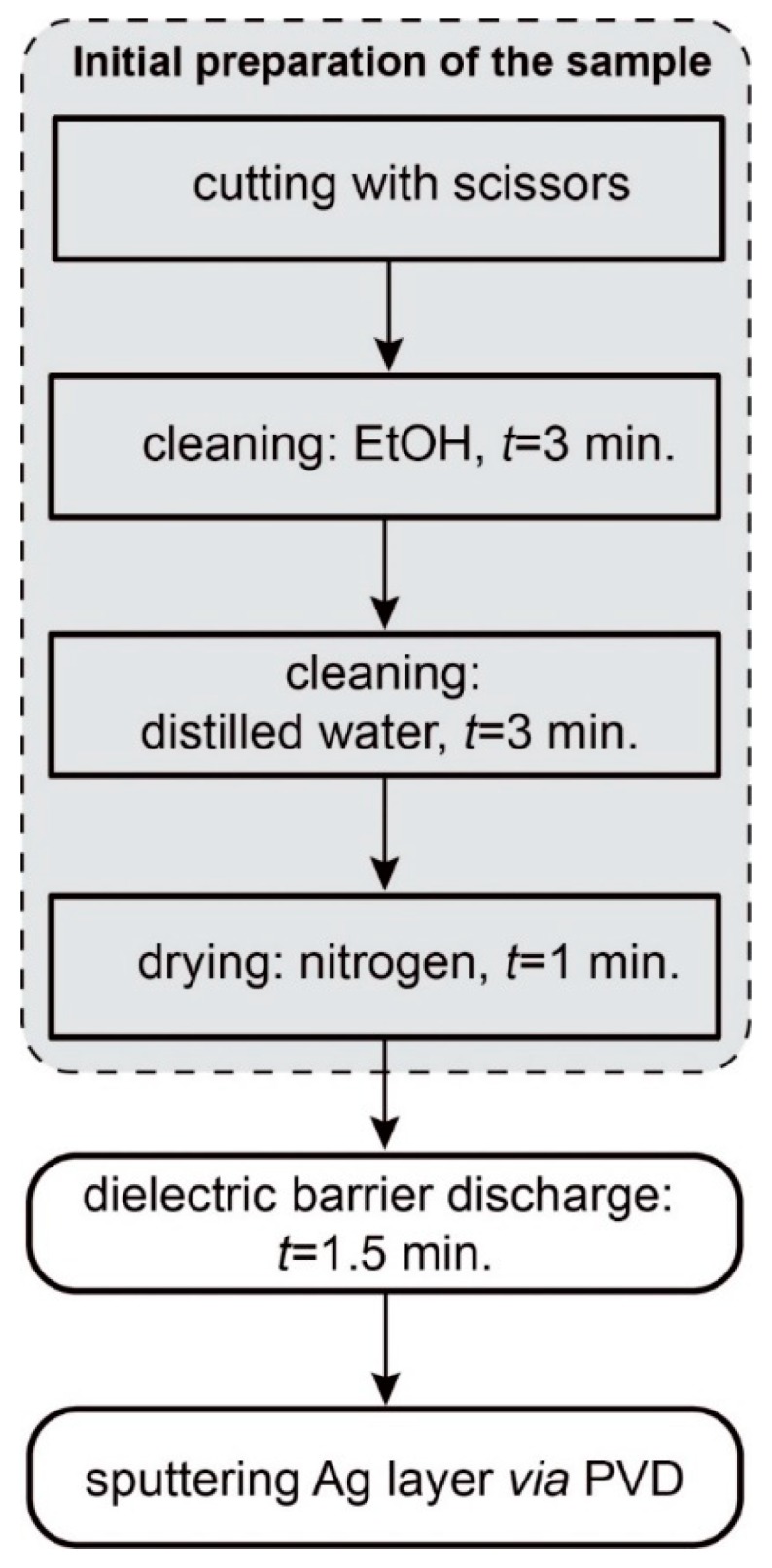
Preparation of the surface-enhanced Raman spectroscopy (SERS) platform consists of three main steps: initial preparation of the sample (cutting, cleaning and drying), modification of the surface layer with dielectric barrier discharge, and finally, sputtering a thin layer of metal with the physical vapor deposition (PVD) method.

Preparation of the SERS platform consists of three main steps listed in Figure 1. Briefly, the initial preparation of the sample consists of cutting and cleaning for three minutes with ethanol (99.8% purity, POCH, Poland) and for three minutes with distilled water and finally drying the sample under a gentle flow of nitrogen (99.9% purity) for one minute. Cleaned samples were placed in a Petri dish and then a dielectric barrier discharge was applied for 90 s (see Section 2.2.1 for detailed description of the setup and method). Immediately after the procedure the sample was sputtered with a layer of SERS-active metal via the physical vapor deposition (PVD) technique (see Section 2.2.2 for details). 

#### 2.2.1. Modification of the Surface via Dielectric Barrier Discharge (DBD) 

**Figure 2:**
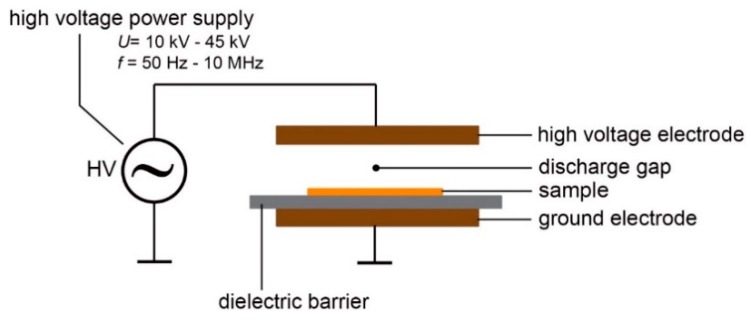
General scheme of the dielectric barrier discharge (DBD) method. The setup consists of two electrodes, the dielectric barrier on or near the one electrode, and the sample between the electrodes. The electrodes are connected to a high voltage power supply, which operates with the voltage between 10 kV and 45 kV and with the frequency between 50 Hz and 10 MHz.

For modification of the surface of the PET/ITO foil a dielectric barrier discharge was used. The general scheme of the apparatus for the DBD is demonstrated in Figure 2. The setup consists of a high voltage (HV) power supply, typically working in the range from 10 kV up to 45 kV, with frequency from ca. 50 Hz up to 10 MHz [60]. The HV power supply was connected to two electrodes (two plates, wires or cylinders), and one of these electrodes was electrically grounded. A dielectric barrier, typically a thin layer of glass, mica, polymer, rubber or ceramics was placed between the electrodes (usually on, or very close to the HV electrode). The sample was placed on the dielectric barrier, so that when HV is applied, the sample is subjected to corona discharge, which modifies the surface of the sample. 

**Figure 3:**
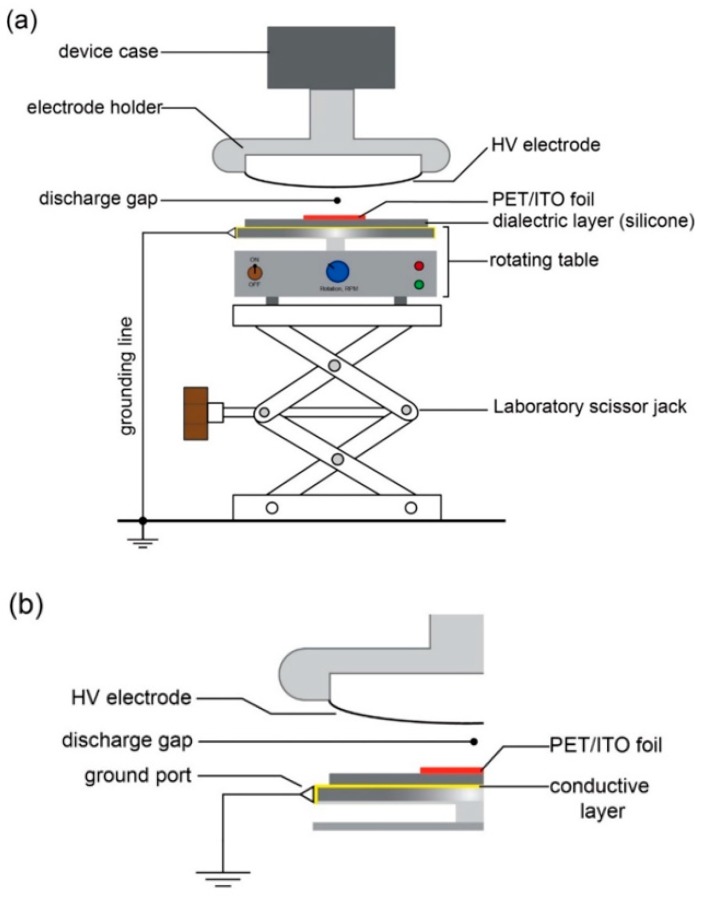
Scheme of the experimental setup for the DBD modification used in our experiments: (**a**) The sample poly(ethylene terephthalate) (PET) covered with a layer of indium tin oxide (ITO) (PET/ITO foil) was placed on a silicone dielectric isolator, which is on the top of a rotating table. The rotation ensures an even and uniform degree of modification of the surface of the sample. The table is placed on the top of a laboratory scissor jack to regulate the distance between the high voltage (HV) electrode and the PET/ITO foil. (**b**) To connect the bottom electrode to the ground, the rotating table was covered with conductive metal layer (copper tape) and a ground port made of an elastic stainless-steel needle was connected with the grounded metal element.

The dielectric barrier discharge modification was conducted with a hand-held laboratory corona treater (ETP, type: BD-20AV, Chicago, IL, USA), equipped with standard three inch wire field-effect electrode. The device has adjustable output voltage in the range of 10 kV to 48 kV and a frequency of 4.5 ± 0.5 MHz [62]. The scheme’s experimental setup is shown in Figure 3a, and a picture of the setup is shown in Figure S1. The corona treater was held in the clamp and placed over a laboratory scissor jack (lab-jack). This solution allows a quick and easy change of the distance between the sample and high voltage electrode. On the top of the lab-jack we placed a home-made rotating table, which allowed speed control. The use of the rotating table made a uniform and repeatable modification of the surface of the sample. The table consists of a housing containing a DC motor, electronics and a rotating head, which is covered with a thin conductive layer of copper. We connected a thin stainless-steel needle as a ground port: This allows to the connection of the rotating head to the ground via copper wire (see Figure 3b). On the rotating head we placed a silicone dielectric layer (1.5 mm thick) and the sample: a 10 mm × 10 mm piece of PET/ITO foil, initially prepared with the procedure described in Section 2.2. 

Finally, we have evaluated the parameters of DBD and set optimal values for our experiments. The parameters we were able to vary were:

(i) the distance *d* (mm) between the high voltage (HV) electrode and the PET/ITO foil,
(ii) the voltage *U* (V) between the HV electrode and the ground,
(iii) the time *t* (s) of the process.

The parameters of the DBD process were chosen based on the series of experiments, where the distance between the PET/ITO surfaces and HV electrode together with the time of using DBD modification and high voltage were evaluated. When the distance, voltage or time were too low, the film surface did not change and the SERS spectrum looked like the spectrum of the film without DBD. However, when the distance, voltage or time were too high, the DBD discharge burned the film, and a discontinuity was formed. The task was to maintain a balance between these three parameters. Optimal conditions were selected using the test method to modify the entire foil surface in the shortest possible time without causing distortion or perforation. To reduce the number of parameters we have chosen the voltage as a constant.

The corona treater is a Tesla coil and there is an easy way to assess the maximal value of the voltage. A nearly linear relationship exists between spark length and spark voltage. A 25 mm spark represents peak voltages of approximately 45 kV, and a 13 mm spark represents a proportional 25 kV. We set the length of the spark to its maximal value, thus the voltage between the HV electrode and ground was set to 45 kV. Such a voltage was used in our all experiments, whereas we varied the distance and the time of modification.

As a result, the voltage was set to *U* = 45 kV, the distance between the sample and HV electrode was set to *d* = 11 mm and the time of modification for *t* = 90 s. These parameters were used for preparation of all SERS platforms. 

#### 2.2.2. Developing of SERS Platform via PVD

To sputter a layer of silver on the top of modified PET/ITO foil, we used the PVD equipment (Quorum, Q150T ES, Laughton, UK). The silver target was obtained from Mennica Metale Szlachetne (Warsaw, Poland). The target diameter was 58 mm, thickness was 0.25 mm, and silver purity was 4 N. The vacuum during the silver sputtering was on the level of 10^−2^ mbar, whereas the sputtering current was 25 mA. The thickness of the sputtered layer was controlled with an embedded quartz microbalance. After the deposition process SERS platforms were placed into a sterile Petri dish.

### 2.3. SERS Measurements

Measurements were carried out with a Renishaw inVia Raman system (Wotton-under-Edge, Gloucestershire, UK) equipped with a 785 nm diode laser. The light from the laser was passed through a line filter and focused on a sample mounted on an X–Y–Z translation stage with a 20× microscope objective, NA = 0.25. The beam diameter was approximately 2.5 µm. The laser power at the sample was 5 mW or less. The microscope was equipped with 1200 grooves per mm grating, cutoff optical filters, and a 1024 × 256 pixel Peltier-cooled RenCam CCD detector (Wotton-under-Edge, Gloucestershire, UK), which allowed registering the Stokes part of the Raman spectra with 5–6 cm^−1^ spectral resolution and 2 cm^−1^ wavenumber accuracy. 

The experiments were performed at ambient conditions using a back-scattering geometry. Typically five spectra were acquired for one chosen pesticide concentration across the PET/ITO/Ag SERS platform. For every sample, based on these five origin spectra, the average spectrum was calculated and they are further presented in the included figures.

### 2.4. Microscopic Characterization

Scanning electron microscopy (SEM) was used to visualize the morphology of the PET/ITO foils before and after modification and sputtering of the metal layer. SEM observations were performed under high vacuum using the FEI Nova NanoSEM 450 (Hillsboro, OR, USA). The accelerating voltage was from 2 kV to 10 kV. The samples of PET/ITO foils were attached to SEM stabs with carbon tape or silver paint and observed without any additional layer of gold or carbon on the surface.

Atomic force microscopy (AFM) measurements were used for topography characterization of the initial foil (AFM, NTEGRA, NT-MDT, Moscow, Russia). The standard non-contact silicon cantilevers (series: ‘Golden’, type: NSG 10) operated in tapping mode were used. The cantilever tip height was 14–16 µm with typical tip curvature radius below 10 nm. All of the samples were measured at the room temperature with the scan rate about 1 Hz. Samples were characterized by parameter of mean roughness Ra, for area 15 μm × 15 μm, that can be expressed as the average deviation of the profile from a mean line for a cross section of the sample topography, using Gwyddion 2.53 analysis software.

The photographs were taken with a digital single-lens reflex (DSLR) camera (80D, Canon) and various lenses (Canon 40 mm/2.8, Canon 50 mm/1.8 and Tamron 90 mm/2.8 Macro). RAW files and SEM pictures (tiff, 16 bits) were developed with ON1 Photo RAW software (ON1, Portland, OR, USA).

### 2.5. Preparation of the Samples for In Situ Detection of Pesticides

Pure pesticide initial solution of 500 µg/mL for Thiram (using acetonitrile/water mixture, 1:1, *v*/*v*) and Carbaryl (in methanol) were prepared. For the Thiram series of standard dilutions, mainly 10, 8, 6, 4, 2, 1 and 0.5 µg/mL were prepared, while for the Carbaryl, the solutions of 100, 80, 50, 30, 10 µg/mL pesticide were chosen. For SERS measurement each sample dilution was placed by drop-coating onto the PET/ITO/Ag platform and then dried at room temperature. All manual operations needed for sample preparation were carried out in the laminar chamber.

The apple juice was prepared shortly before experiments. The 2 µL of each pesticide standard solution was added to 100 µL of the apple juice. The mixture was vortexed for one minute and centrifuged at 10,000 rpm for 10 min. Then the supernatant (10 µL) was directly placed on the as-prepared PET/ITO/Ag SERS platform and dried at room temperature. Additionally, the pesticide concentrations on the apple skin were detected at the maximum residues limit [63]. The solution concentration of 5 µg/mL for Thiram and 0.01 µg/mL for Carbaryl pesticides was spread onto apple skin, and then the flexible platform was applied. The contact time between the SERS platform and apple was 60 s. The platforms were placed under the microscope and measurements were performed with the parameters described in Section 2.3.

## 3. Results and Discussion

### 3.1. Morphology of the Platform

**Figure 4:**
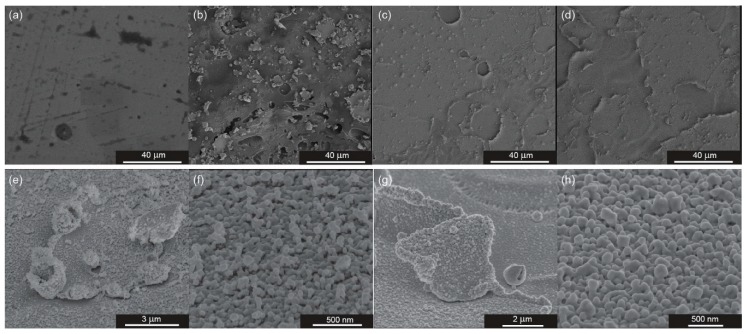
Scanning electron microscopy (SEM) images of PET/ITO foil in few different states of SERS platform preparation: (**a**) raw PET/ITO foil without any modifications, (**b**) foil after 90 s of the DBD, (**c**) foil modified with DBD and with 30 nm layer of silver, (**d**) foil modified with DBD and with 70 nm layer of silver, (**e**,**f**) images of foil modified with DBD and with 30 nm layer of silver at high magnifications, and (**g**,**h**) images of foil modified with DBD and with 70 nm layer of silver at high magnifications.

A dielectric barrier discharge plasma, operating in air at atmospheric pressure, has been used to induce changes in the surface morphology of polyethylene terephthalate (PET) foil with an indium tin oxide (ITO) layer. Moreover, the morphology of the raw PET/ITO foil was examined with AFM (see Figure S3, Supplementary Materials). The cross-section of the PET/ITO/Ag platform made with SEM is demonstrated in Figure S4 (Supplementary Materials) and confirms that thickness of the PET/ITO composite is 130 µm. SEM pictures taken in different stages of SERS platform preparation are presented in Figure 4.

PET/ITO foil without any modification (Figure 4a) has a very uniform morphology, with Ra = 7 ± 1 nm (Figure S3). Due to large changes in surface topography that exceeded the research capabilities of the AFM microscope, only AFM images obtained for the initial PET/ITO foil are presented. As is presented in Figure 4b–d, the continuous PET/ITO layer is largely modified by DBD. After the sputtering of silver, the surface consists of various inconsistencies, which largely influences the existence of the enhancing properties. SEM images at large magnification are presented in Figure 4e,f for PET/ITO after DBD modification and sputtering of 30 nm of silver, and Figure 4g,h for PET/ITO after DBD modification and sputtering 70 nm of silver. The morphology of the samples depends on the scale: DBD makes the surface developed and non-regular, whereas at nanoscale the surface is very uniform with objects of diameter from 50 nm to 100 nm. The Figure S5 and S6 (Supplementary Materials) shows the PET/ITO foil covered with 30 nm and 70 nm of silver, but without DBD treatment. The structure is very flat and corresponds with the morphology of the ITO layer, with objects barely seen on the surface of the foil. Rare, big objects come from non-uniformity of the ITO layer. The flat morphology is responsible for a very low enhancement factor (see Figure S7, Supplementary Materials). Analysis of the SEM images demonstrate that DBD treatment causes damage of the surface of the foil, especially the surface of ITO. The damage is in scale of micrometers (large non-uniformities) and single nanometers. During the sputtering of silver via the PVD method these nanometric non-uniformities work as a centers of crystallization, where silver aggregates and produces bigger clusters (see picture Figure 4f,h).

### 3.2. Enhancing Properties of Fabricated Platform

**Figure 5:**
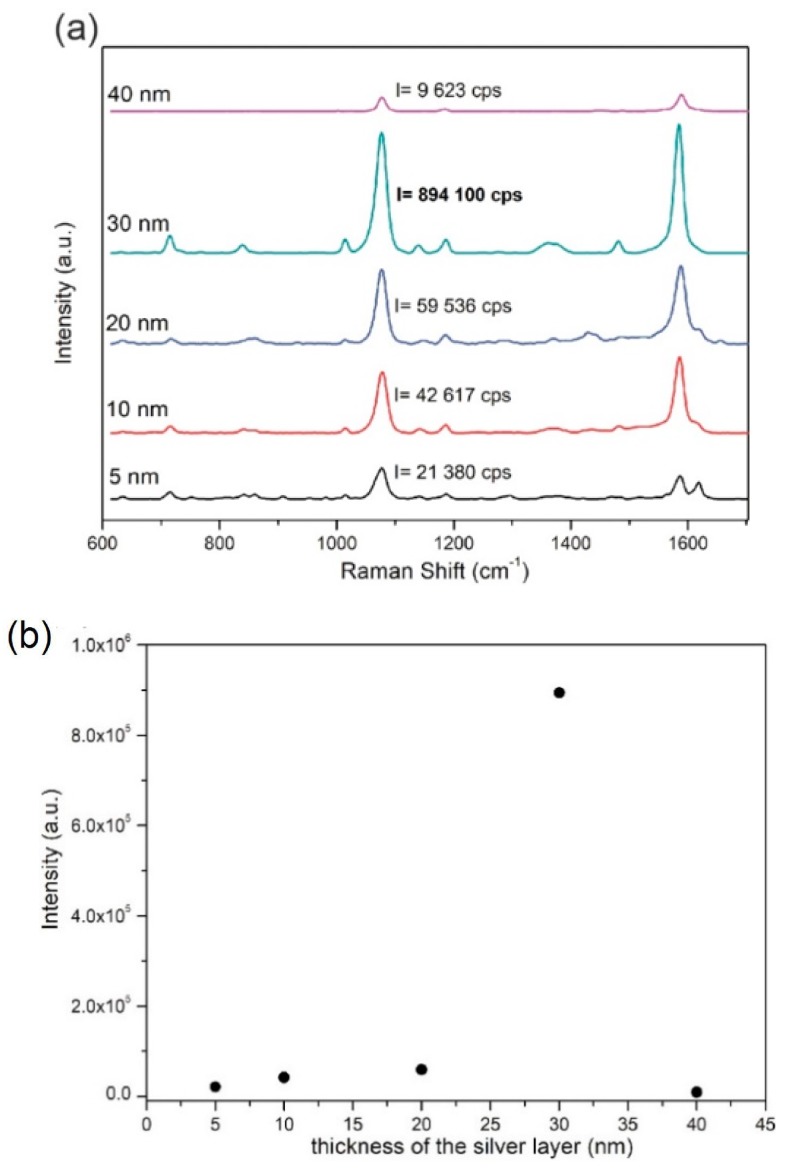
SERS spectra of *para*-mercaptobenzoic acid (*p*-MBA) (10^−6^ M) on PET/ITO/Ag platforms with various silver thicknesses. (**a**) Intensity of a band at 1075 cm^−1^ in relation to sputtered silver thickness. (**b**) SERS spectra were recorded using a 785 nm laser excitation (the laser power was below 5 mW) with three accumulations for four seconds.

The maximal enhancing properties of fabricated PET/ITO/Ag SERS platforms were optimized by applying different thicknesses of the sputtered silver layer, i.e., 5, 10, 20, 30 and 40 nm. Freshly prepared platforms were soaked for 24 h with *p*-MBA (10^−6^ M) and afterwards the SERS experiments were performed. In Figure 5a the presented SERS spectra are dominated by the 1077 cm^−1^ band, which was selected to find the optimal thickness of the sputtered silver layer. Intensity at this band presented as a function of the thickness of the silver layer (Figure 5b) shows that the largest gain (894 100 counts) occurs for a thickness of the silver layer of 30 nm. Therefore, the 30 nm silver value was chosen and used throughout presented experiments.

In order to increase the importance of the DBD modification on the enhancing properties of fabricated platforms, additional SERS experiments were performed in the mapping mode over the two differently prepared PET/ITO/Ag platforms, with and without DBD modification. Both platforms were covered with a 30 nm Ag layer and then platforms were immersed overnight in a 10^−6^ M solution of the *para*-mercaptobenzoic acid chosen as standard. The obtained results clearly indicate the importance of DBD modification, as the platforms without DBD modification show very low enhancing properties. In Figures S7 and S8 (Supplementary Materials) the maximum intensity signal at 1075 cm^−1^ reaches 250 cps, while the signal gathered using the DBD modified platform reaches over 5 × 10^5^ cps. 

Additionally, the enhancement factor (EF) [64,65] was calculated based on the SERS and normal Raman spectrum gathered for standard solution and crystals of *p*-MBA, respectively. The representative, intensive band at 1077 cm^−1^ was selected to calculate the EF. The SERS signal intensity at 1077 cm^−1^ is 2.5 × 10^5^ cps, while normal Raman signal intensity at 1096 cm^−1^ is 502 cps (cps taken from averages spectra presented on Figure S7, Supplementary Materials). We calculated the EF according to information given in the Supplementary Materials. The calculated data gives EF value of 2 × 10^6^. This high value of the enhancement factor is related to the morphology of the samples at the nanoscale. Figure 4e–h demonstrates the PET/ITO foil after DBD and sputtering of 30 nm or 70 nm of silver at high magnifications. On the nanoscale level the samples possess a highly developed and uniform structure with silver objects/aggregates of diameter from 50 nm to 100 nm. The size is in a good agreement with the size optimal for plasmon resonance, thus the platforms ensure an enhancement factor of the order of 10^6^. At the same time, we obtain very low level of SERS signal (200 cps) for the sample covered with silver layers (both 30 nm and 70 nm) without previous DBD treatment. The high magnifications (Figures S5 and S6, Supplementary Materials) reveal that silver layers do not form any clusters and aggregates suitable for enhancement of SERS signal on the raw PET/ITO layer. Also, the high homogeneity of SERS enhancement (Figure S7a, Supplementary Materials) is observed despite of the fact that the SERS platform is non uniform at the micro-scale (see Figure 4c,d).

### 3.3. SERS Spectra of Pesticides

**Figure 6:**
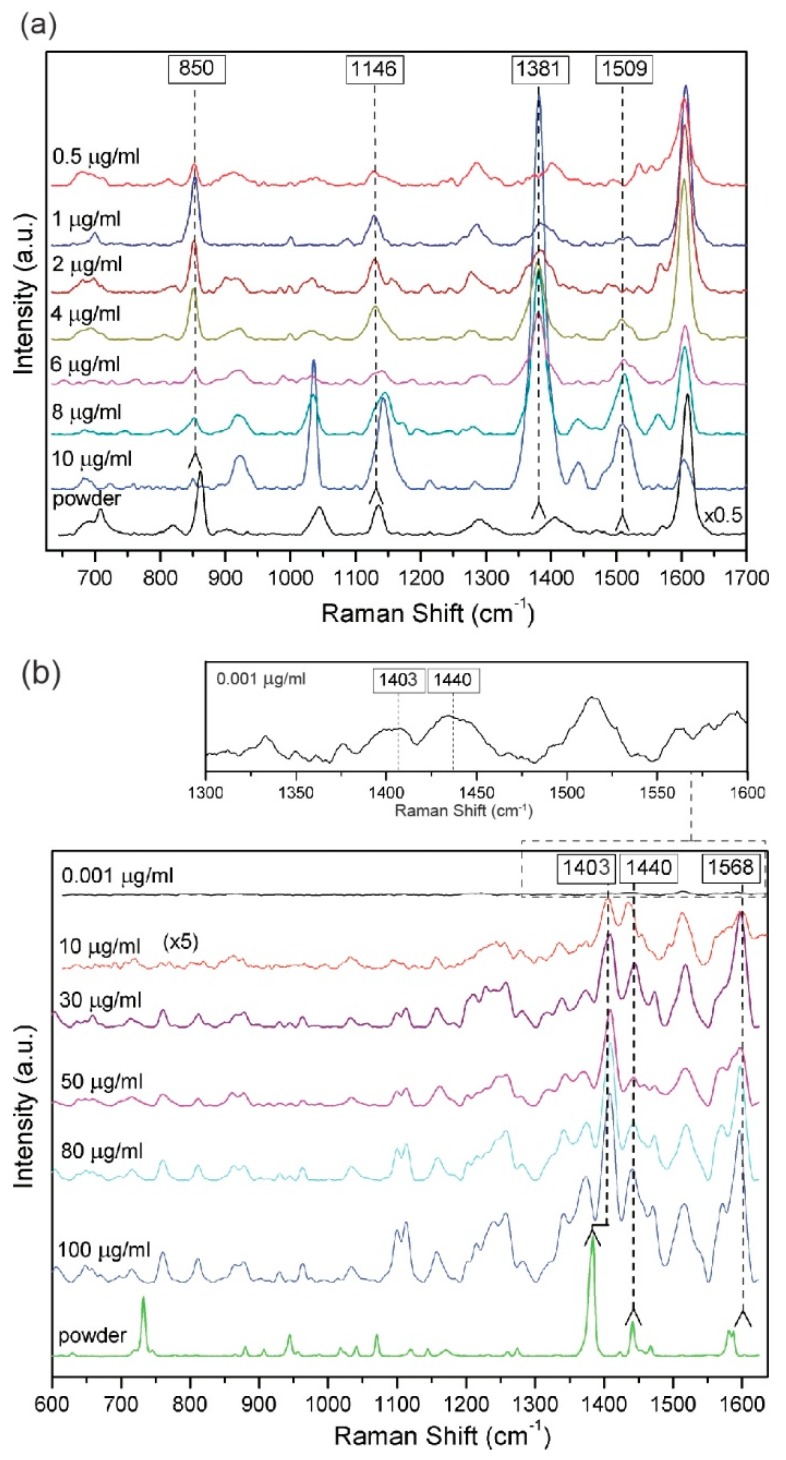
The averaged selected SERS spectra of Thiram (**a**) and Carbaryl (**b**) pesticides, measured in five different places of PET/ITO/Ag platforms. The Raman spectra of pesticides powder are presented on the bottom of each figures. The main Raman bands are indicated by dashed lines. Three accumulation for four seconds of SERS spectra were recorded using a 785 nm laser excitation (the laser power was below 5 mW).

Two pesticides studied in the framework of this paper, i.e., Thiram and Carbaryl differ structurally as can be seen in Figure S1 (Supplementary Materials). Due to structural differences also the changes in a SERS spectrum of these pesticides in relation to the platform surface and pesticide interaction are expected. Figure 6 presents SERS spectra taken for different, chosen concentrations of each pesticide gathered together with the Raman spectra of pure pesticides. The pesticides concentrations were chosen relaying on information concerning those chemical compounds, e.g., the maximum residue limit (MRL) of Carbaryl is 0.01 mg/kg, whereas the MRL for Thiram is set to 5 mg/kg [63]. SERS spectra presented for Thiram in Figure 6a show four the most prominent bands characteristic for vibration modes of CH_3_N stretching, CH_3_ rocking and/or CN stretching, CH_3_ deformation (at 850, 1146 and/or 1381, 1406 cm^−1^, respectively). The most intensive band at 1381 cm^−1^ indicates the importance of S-S surface metal bonding. Thus, as was expected, due to disulfide S-S bonds this compound chemisorbs dissociatively on the PET/ITO/Ag SERS platform, as was already observed [66]. The behavior of the band intensity at 850 cm^−1^ is a bit more complex than observed intensity changes in the case of other bands. The band at 850 cm^−1^ is characteristic for vibration of CH_3_N stretching. With decreasing of the concentration of Thiram to 6 µg/mL the intensity of the band at 850 cm^−1^ is also decreasing, while for the region 4 µg/mL–1 µg/mL of pesticide concentration, increasing of this mode appeared, and again with further decreasing of the concentration the decreasing of its intensity is detected. This behavior is probably influenced by differences in the molecular alignment of the analyte in relation to the surface of the platform. With decreasing of the analyte concentration the molecular alignment is changing and likely only one molecular layer is covering the surface of the platform. In such a condition this mode can be more intensive. Then again with decreasing of the pesticide concentration the usual intensity–concentration relation is detected. SERS spectra of Carbaryl show three of the most intensive bands at 1403, 1440 and 1568 cm^−1^ strictly related to the symmetric and mono-substituted bond vibrations of naphthalene ring, and stretching of a C=C group, respectively [12] (Figure 6b). The assignments of these bands are gathered in Table 1. Moreover, whole observed spectral features of pure pesticides and their solutions show differences in the sense of observed band intensities. Such a behavior is induced by pesticide interaction with a metal surface of the SERS platform. For both studied pesticides observed in SERS, the spectra bands are in close relation to the bands observed in the Raman spectrum of the pure pesticides. It should be noted, that the Raman spectrum presented for pure Thiram shows differences in comparison to the SERS spectrum of its solution, e.g., the band at 1381 cm^−1^ in the spectra of pure Thiram undergo splitting. Additionally, due to the metal surface and pesticide interaction, some additional vibrational modes have appeared. These can especially be seen in the case of the Raman and SERS spectra of Carbaryl.

### 3.4. Reproducibility of SERS Signal and Calibration Curve of Pesticides

**Figure 7:**
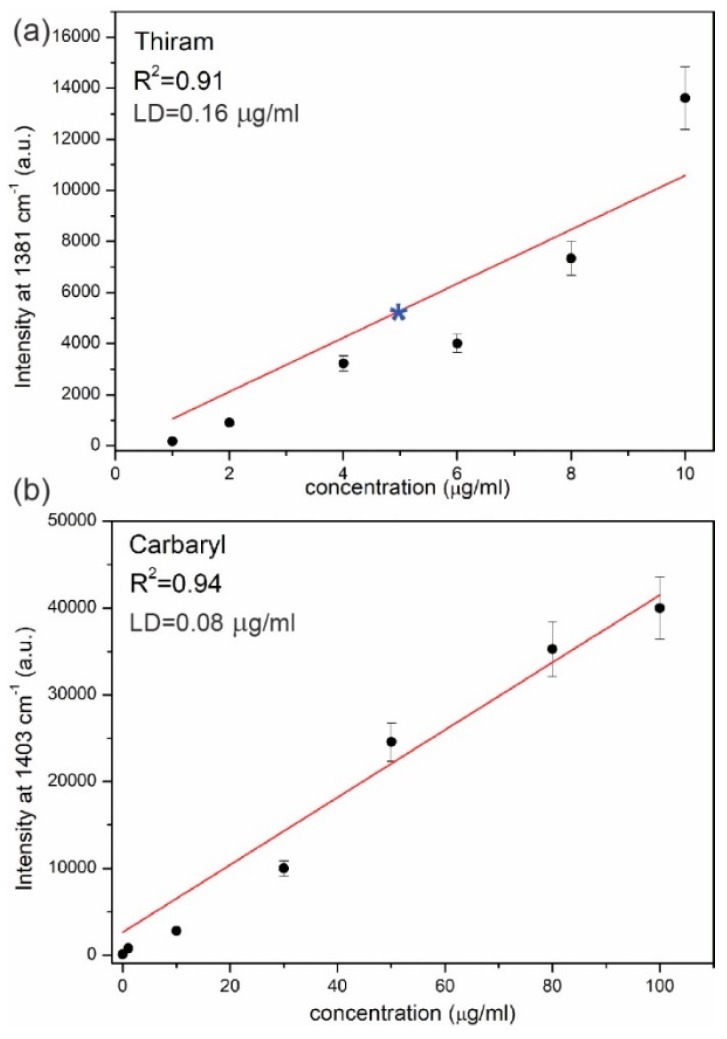
Calibration curves for Thiram (**a**) and Carbaryl (**b**) pesticides. Each point is based on the average SERS spectra taken in five different places on the PET/ITO/Ag platform. The error bars indicates 9% deviation at a chosen band. Star corresponds to the intensity of the 1381 cm^−1^ band in the SERS spectrum of the apple juice with Thiram pesticide. The limit of detection (LD) for Thiram is 0.16 µg/mL, whereas for Carbaryl it is 0.08 µg/mL.

**Figure 8:**
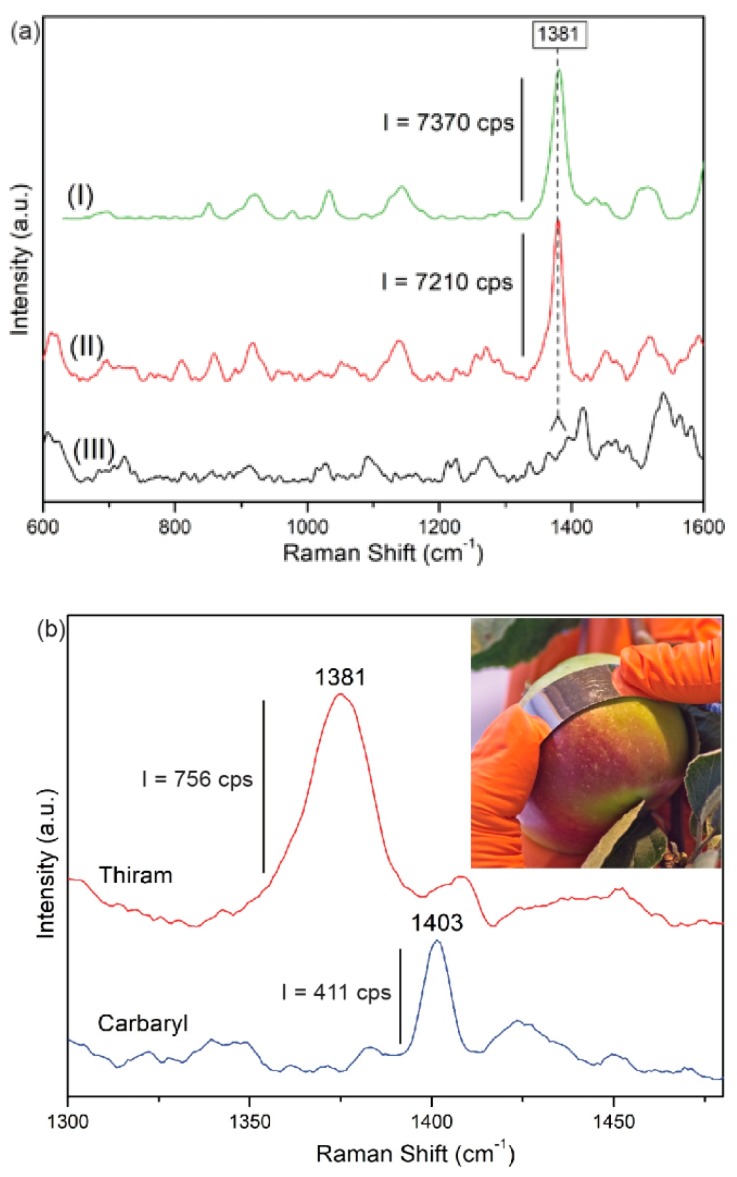
(**a**) SERS spectra on PET/ITO/Ag platform recorded for Thiram pesticide solution (I), Thiram in apple juice (II), and apple juice (III), and (**b**) the detected traces of both Thiram and Carbaryl taken from an apple’s skin. The inset presents photo of pesticide collection from an apple’s skin. SERS spectra were recorded using a 785 nm laser excitation with the laser power below 5 mW with three accumulations for four seconds.

For both pesticides, the reproducibility of SERS signals obtained for presented platforms were also investigated. The SERS spectra of each pesticide concentration were collected in five different places across the platform. The intensity differences among the spectra at the chosen band at 1381 cm^−1^ for Thiram, and at 1403 cm^−1^ for Carbaryl, oscillating at about 9%. For both studied pesticides gathered data reveals a linear relationship between the intensity at the chosen point and the pesticides’ concentration (Figure 7a). Presented data include a 9% error bar at each average point. The obtained linear curve provides a calibration for quantitative detection of Thiram and Carbaryl pesticides in food. The accuracy of the obtained fellowship for Thiram was checked using apple juice with a given pesticide concentration. Figure 8a presents spectra of apple juice, apple juice with a given Thiram concentration, and Thiram for comparison. As can be seen the similar intensity of the band at 1381 cm^−1^ for both mixtures, apple juice with Thiram and the corresponding concentration of Thiram solution was obtained. In the Thiram calibration curve the intensity of this band in the SERS spectrum of apple juice with Thiram is included as a star point (see Figure 7a). The excellent agreement between the applied concentration of the Thiram pesticides in the apple juice and the gain in intensity of the 1381 cm^−1^ band was obtained. This result indicates that Thiram pesticide can be quantified by SERS using fabricated PET/ITO/Ag platforms based on the calibration curve. Therefore, in the next step the calibration curves were adopted for trace analysis of pesticides directly from the skin of an apple (Figure 8b). In this experiment, the pesticide concentrations on the skin of an apple were applied as 5 µg/mL and 0.01 µg/mL (Thiram and Carbaryl, respectively) according to the maximum residue limits (MRL) [63]. As can be seen in Figure 8b, the most prominent marker bands at 1381 cm^−1^ and 1403 cm^−1^ corresponding to Thiram and Carbaryl, respectively were recorded. Additionally, the limit of detection (LD) were calculated for both pesticides with the equation:

$$LD=3.3\cdot SD\cdot a^{-1}$$

where *SD* is the standard deviation of the Y-intercept and a is the slope of linear fit. The *LD* for Thiram is equal to 0.16 µg/mL and for Carbaryl is 0.08 µg/mL.

Based on the constructed calibration curves (Figure 7) the amount of the pesticides from the fruit skin were estimated as 2.5 µg/mL for Thiram and 0.012 µg/mL for Carbaryl. Presented results demonstrate the potential of PET/ITO/Ag flexible SERS platform for quantitative analysis of pesticides in the field.

## 4. Conclusions

A low-cost, flexible PET/ITO/Ag SERS platform with optimized silver thickness were fabricated using a method combining the dielectric barrier discharge (DBD) and physical vapor deposition (PVD) technique. These platforms show a high enhancement factor calculated to be 2 × 10^6^ in comparison to normal Raman data. The PET/ITO/Ag with 30 nm of silver layer was selected as the optimal for the pesticide detection. Fabricated flexible PET/ITO/Ag SERS platform and constructed calibration curves enable the quantitative SERS determination of Thiram and Carbaryl pesticides concentration in the skin of apple. The estimated concentrations of the pesticides equals 2.5 µg/mL and 0.012 µg/mL for Thiram and Carbaryl, respectively. Therefore, the spectroscopic properties of the developed SERS platform allows the detection of the pesticides up to the maximum residue limit (MRL). The value of 2.5 µg/mL is below the MRL (the apple can be introduced to the market), while the second is just a little above the MRL value (harmful fruit). All these data indicate that the proposed approach of SERS platform preparation provides high signal enhancement that enables label-free, reproducible, and sensitive detection of pesticides. It is expected, that the PET/ITO/Ag platform will find broad application in various environmental toxicants analysis.

## Figures and Tables

**Table 1:** The band assignments of major peaks in SERS spectra for Thiran and Carbaryl [11,49].

Pesticide	SERS Raman Shift (cm^−1^)	Band Assignment
Thiram	860	υ(CH_3_N)
	921	υ(CH_3_N), υ(C=S)
	1142	ρ(CH_3_), υ(CN)
	1381	δs(CH_3_), υ(CN)
	1444	δas(CH_3_), υ(CN)
	1508	υ(CN), δ(CH_3_), δas(CH_3_)
Carbaryl	1403	symmetric ring vibration naphthalene
	1440	unspecified ring vibration of mono-substituted naphthalene
	1568	υ(C=C) in naphthalene ring

υ—stretching, δ—deformation, ρ—rocking, s—symmetric, as—asymmetric.

## Supplementary Materials

The following are available online at https://www.mdpi.com/2079-6374/9/3/111/s1.
