# Supplementary material for: Flexible PET/ITO/Ag SERS Platform for Label-Free Detection of Pesticides
Source: Biosensors (Basel). 2019 Sep 19;9(3):111. doi: 10.3390/bios9030111 (PMC6784364; doi:10.3390/bios9030111)
Supplement: Supplementary file 1 [file biosensors-09-00111-s001.pdf]

Electronic Supporting Information

Flexible PET/ITO/Ag SERS platform for label-free detection of pesticides

Ariadna B. Nowicka, Marta Czaplicka, Aneta Kowalska, Tomasz Szymborski*,
and Agnieszka Kamińska

*corresponding author, tszymborski@ichf.edu.pl

Institute of Physical Chemistry, Polish Academy of Sciences, Kasprzaka 44/52,

01-224 Warsaw, Poland

	Page
**Figure S1** Chemical structure of the pesticides used in the experiments: Thiram (a) and Carbaryl (b)	2
**Figure S2** Picture of the experimental setup (a) and dielectric barrier discharge between the High Voltage electrode and PET/ITO substrate (b).	3
**Figure S3** AFM of the pure PET/ITO foil (a) 2D view and (b) 3D view, roughness of the surface (c).	4
**Figure S4** Cross-section of the PET/ITO/Ag foil made with SEM.	5
**Figure S5** Scanning Electron Microscope (SEM) images of the PET/ITO foil without DBD and covered via PVD with a 30 nm layer of silver.	6
**Figure S6** Scanning Electron Microscope (SEM) images of the PET/ITO foil without DBD and covered via PVD with a 70 nm layer of silver.	7
**Figure S7** SERS data recorded in the mapping mode (20 µm x 20 µm) for p-MBA (10^{-6} M) on the PET/ITO/Ag platform prepared with DBD modification (a) and without such activation of the platform surface (b).	8
**Figure S8** SERS spectra of *p*-MBA (10^{-6} M) recorded on the PET/ITO/Ag platform prepared with DBD modification (red line) and normal Raman spectrum recorded for p-MBA crystal (black line).	9
**The SERS enhancement factor (EF) calculation**	10

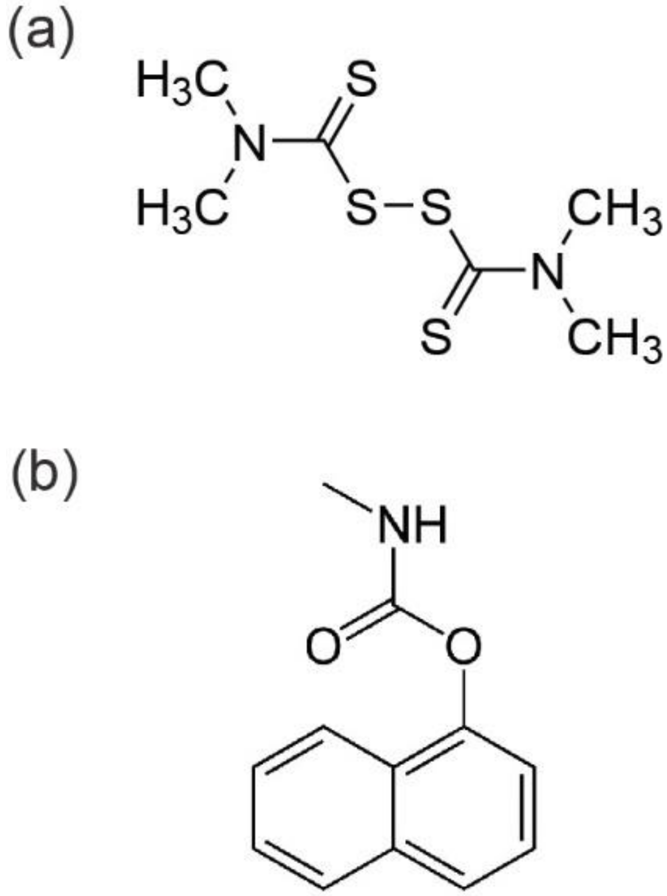

Figure S1 Chemical structure of the pesticides used in the experiments: Thiram **(a)** and Carbaryl **(b)**

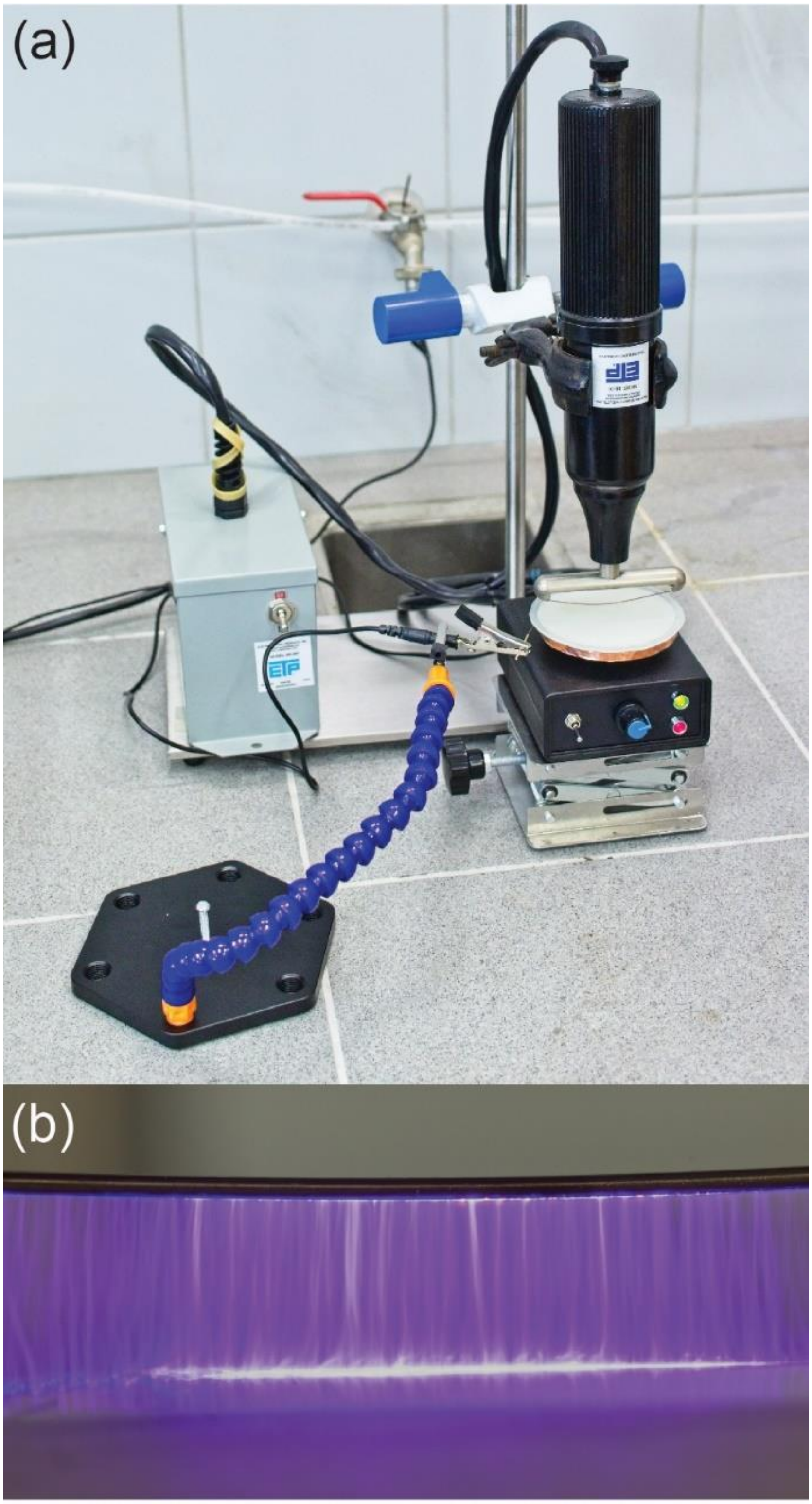

Figure S2 Picture of the experimental setup **(a)** and dielectric barrier discharge (DBD) between the High Voltage electrode and PET/ITO substrate **(b)**.

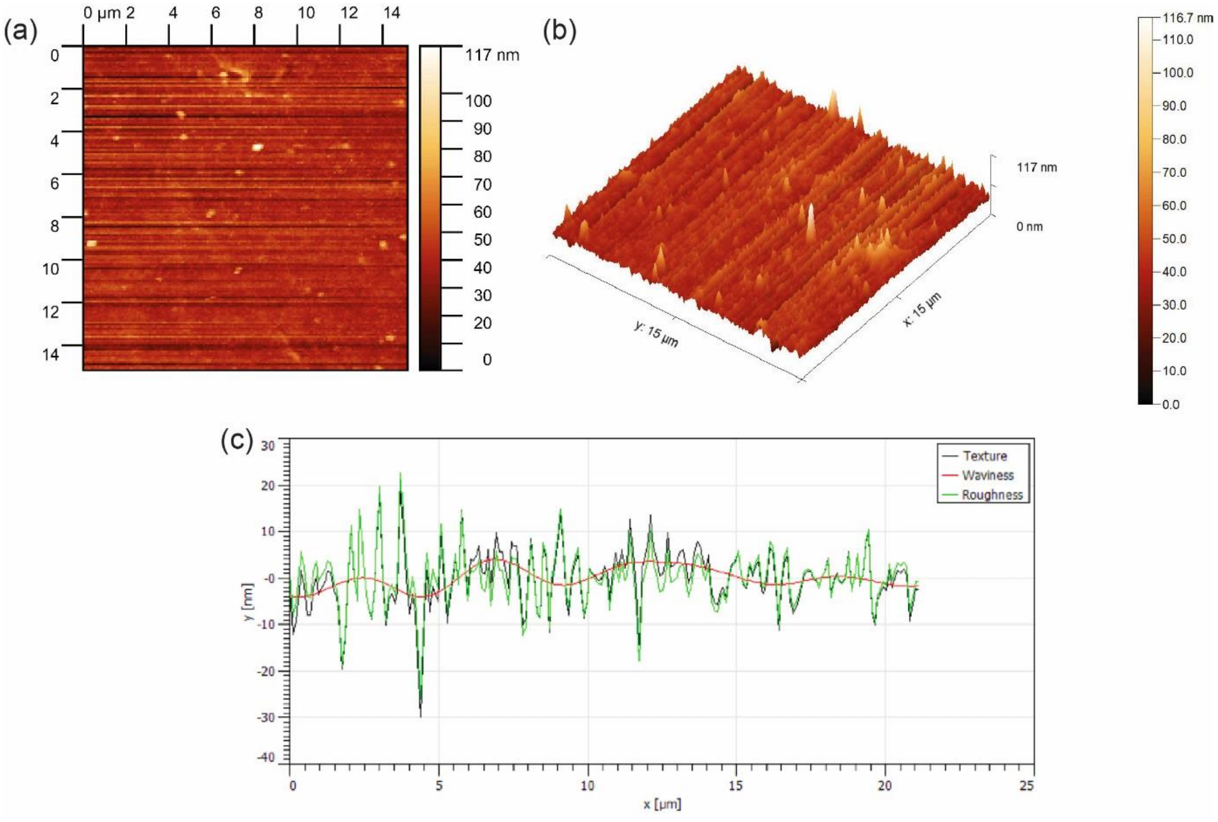

Figure S3 AFM of the pure PET/ITO foil **(a)** 2D view and **(b)** 3D view, roughness of the surface, Ra=7±1 nm **(c)**.

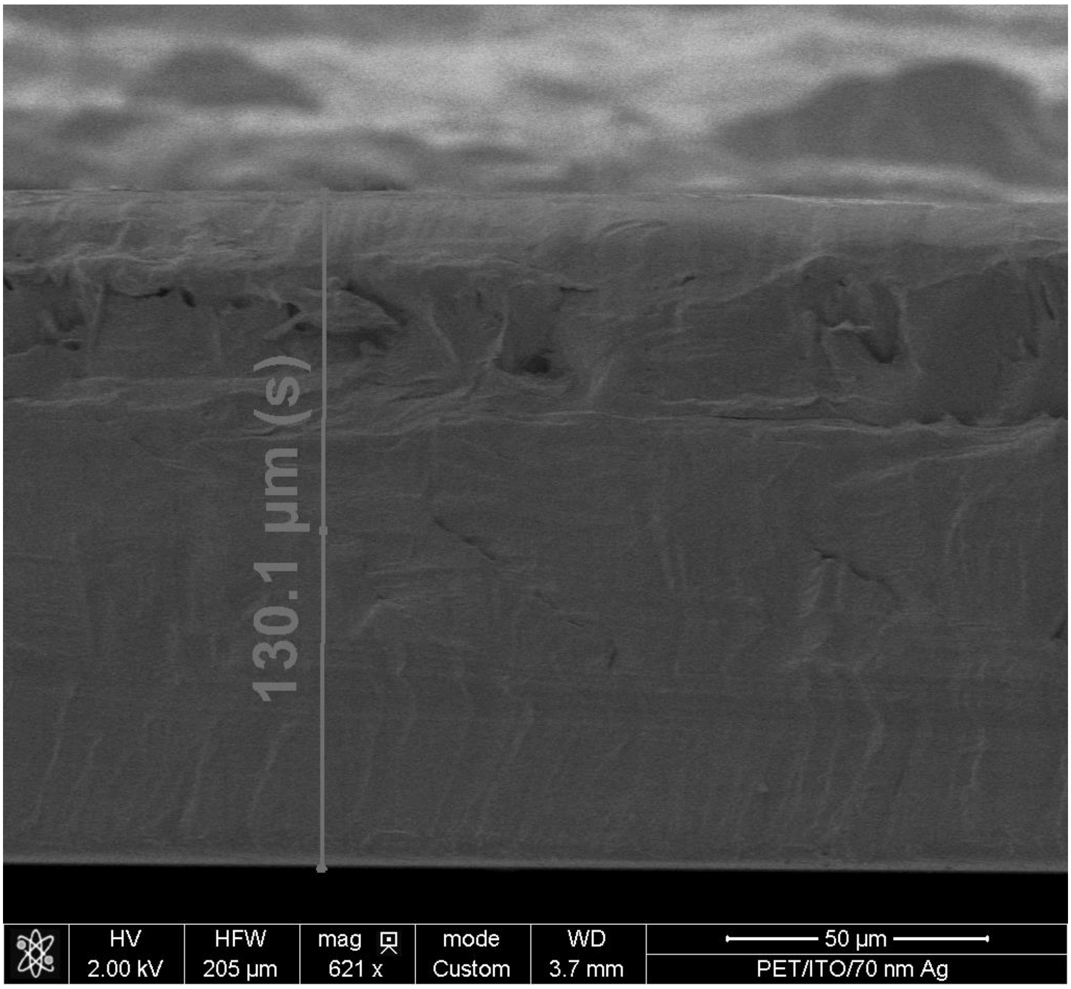

Figure S4 Cross-section of the PET/ITO/Ag foil made with SEM. The measured thickness of the composite is 130 µm (125 µm of PET foil and 5 µm of ITO layer). The thickness of the silver layer is too small (1/2000 of the total thickness of PET/ITO foil) to clearly measure it with Scanning Electron Microscope.

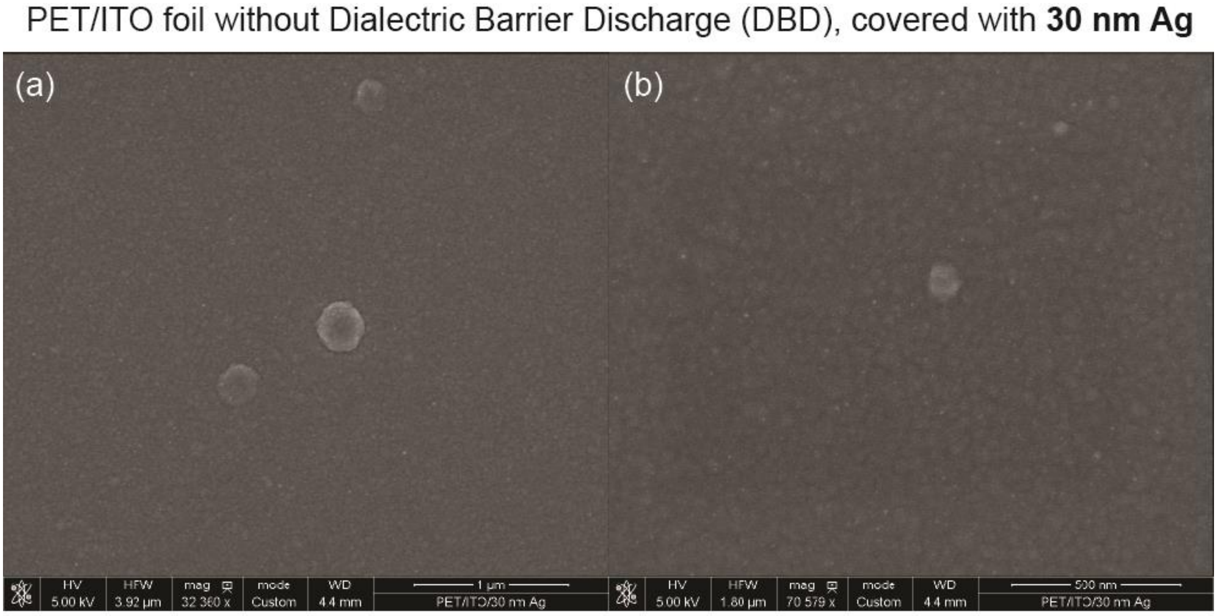

Figure S5 Scanning Electron Microscope (SEM) images of the PET/ITO foil covered *via* PVD with a 30 nm layer of silver. The sample was not subjected to dielectric barrier discharge (DBD). The morphology of the layer of silver is a reflectance of the structure of ITO layer.

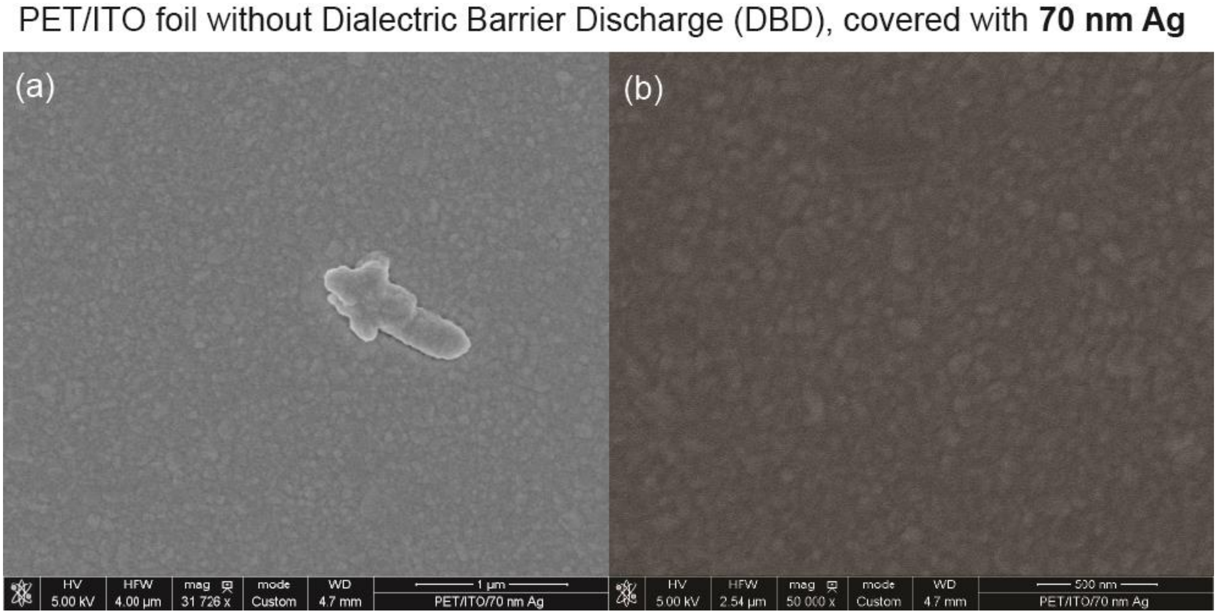

Figure S6 Scanning Electron Microscope (SEM) images of the PET/ITO foil covered via PVD with a 70 nm layer of silver. The sample was not subjected to dielectric barrier discharge (DBD). The morphology of the layer of silver is a reflectance of the structure of ITO layer.

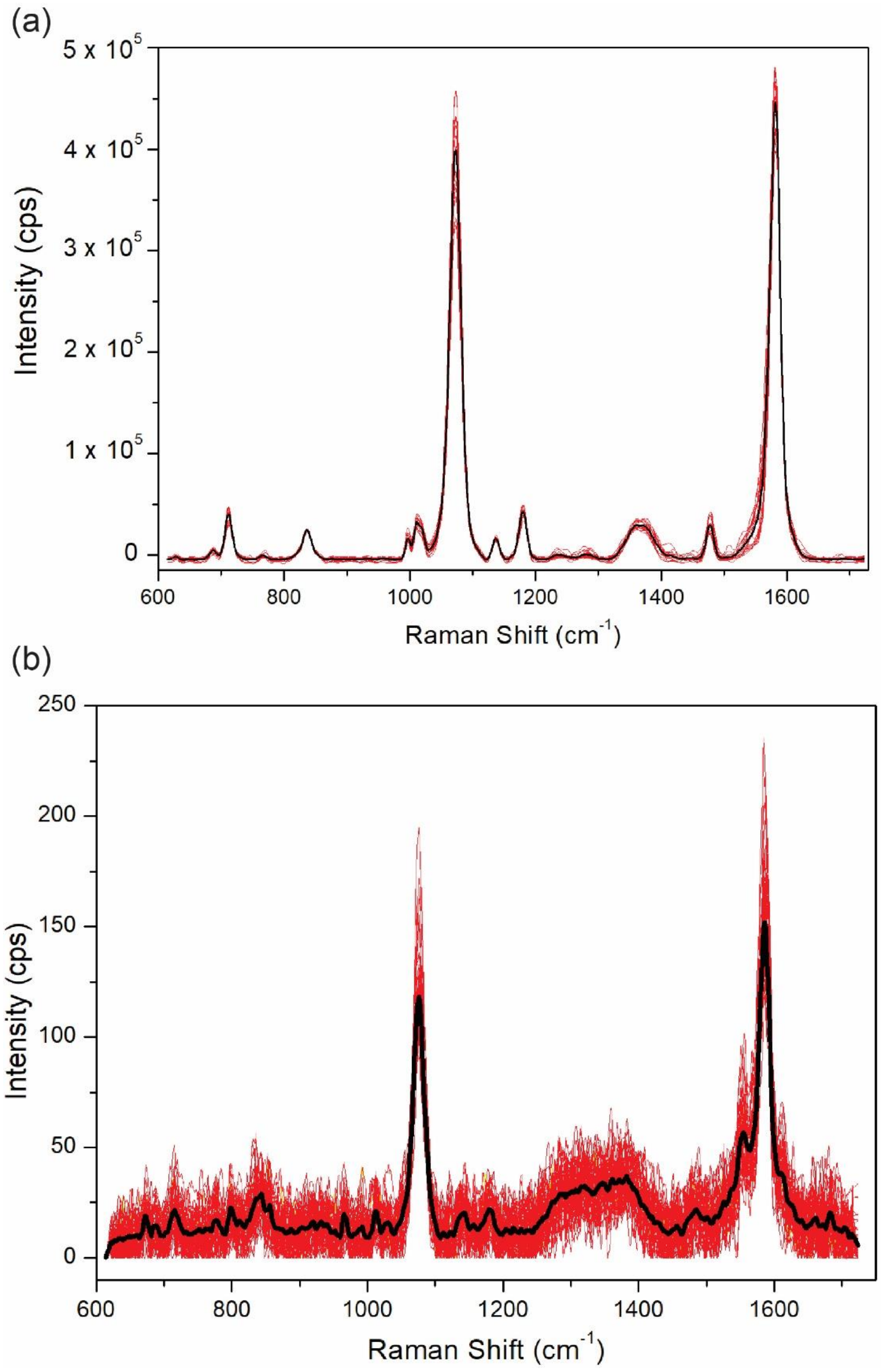

Figure S7 SERS data recorded in the mapping mode (20 µm × 20 µm) for *p*-MBA (10^{-6} M) on the PET/ITO/Ag platform prepared with DBD modification **(a)** and without such activation of the platform surface **(b).** The black line represents the average spectrum calculated from 40 spectra (red lines).

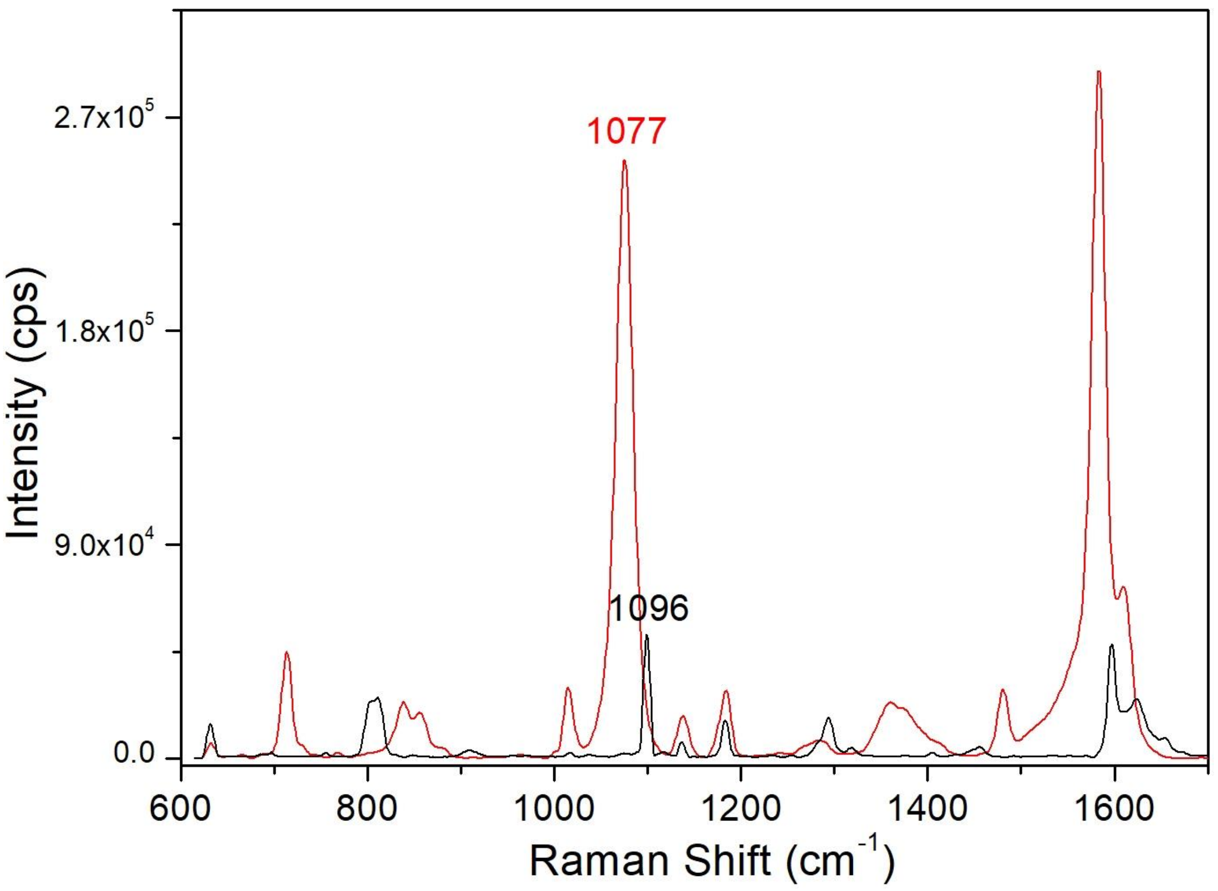

Figure S8 SERS spectra of *p*-MBA (10^{-6} M) recorded on the PET/ITO/Ag platform prepared with DBD modification (red line) and normal Raman spectrum recorded for *p*-MBA crystal (black line). To present both spectra in one figure the normal Raman spectrum (black line) was multiplied by factor ×100.

The SERS enhancement factor (EF) calculation

EF calculation were based on comparison the SERS signal excited the *p*-MBA (10^{-6} M) standard solution adsorbed on the PET/ITO/Ag platform and the Raman signal of powder *p*-MBA sample.

In SERS measurements – the volume of space occupied by single *p*-MBA molecule is 2×10^{-19} m^2.[i]

The PET/ITO/Ag platform (surface 2.5×10^{-5} m^2) were overnight soaked in the 2 mL of 10^{-6} M *p*-MBA. The number of molecules contained in the solution was:
6.02×10^{23} molecules/mol × 2.0×10^{-3} L × 10^{-6} mol/L = 1.2×10^{15} molecules.
This give total area of 2.4×10^{-4} m^2 is occupied by *p*-MBA on the platform (which is smaller than platform), therefore we consider, only one layer of *p*-MBA molecules covers PET/ITO/Ag platform.

The surface area irradiated by the laser beam (2.5 µm in diameter) was 4.9 μm^2 (3.14 × 1.56 μm^2 = 4.9 μm^2). The surface of our samples was 2.5×10^{-5} m^2. Therefore, about 9.8×10^7 molecules were present in the laser beam spot (N_{SERS}).

In the normal Raman measurement – Raman signal were collected form the crystal of *p*-MBA (density of 1.19 g/cm^3). The surface area irradiated by the laser beam (2.5 µm in diameter) was 19.6 x 10^{-8} cm^2 (3.14 × 1.56 μm^2 = 4.9 μm^2). As 785 nm laser beam is able to easily penetrate through every point in solid samples on *ca.* 2 mm depth, therefore the effective illuminated volume for our setup is 3.92×10^{-8} cm^3. This value was confirmed by registering Raman spectra of silicon while varying the distance from the focal plane. Therefore, taking into account the crystal density, the Avogadro number of molecular entities and effective illuminated volume, about 3.8×10^{11} molecules were present in the laser beam spot (N_{Raman}).

Raman equipment - Since SERS and Raman spectra were obtained under same excitation angle, the laser spot area of normal Raman can be ignored (*A* in the equation).

Calculation were based onto following equations:

$$EF = \frac{I_{SERS}}{N_{SERS}} \cdot \frac{N_{Raman}}{I_{Raman}}$$

where:

- N_{Raman} and N_{SERS} denote the number of probe molecules which contribute to the normal and SERS signals,
- I_{Raman} and I_{SERS} denote the corresponding normal Raman and SERS intensities.

$N_{SERS} = M_{SERS}/S_{SERS}$

$N_{Raman} = M_{Raman}/S_{Raman}$

Under these conditions: $N_{SERS} = 9.8 \times 10^{7}$ and $N_{Raman} = 3.8 \times 10^{11}$

[i] Yingying Yu, Saori Handa, Toru Yajima, Masayuki Futamata, *Chemical Physics Letters* 560 (2013) 49–54
